# Pokkali: A Naturally Evolved Salt-Tolerant Rice Shows a Distinguished Set of lncRNAs Possibly Contributing to the Tolerant Phenotype

**DOI:** 10.3390/ijms241411677

**Published:** 2023-07-20

**Authors:** Shalini Tiwari, Mukesh Jain, Sneh Lata Singla-Pareek, Prem L. Bhalla, Mohan B. Singh, Ashwani Pareek

**Affiliations:** 1 Stress Physiology and Molecular Biology Laboratory, School of Life Sciences, Jawaharlal Nehru University, New Delhi 110067, India; 2 School of Computational and Integrative Sciences, Jawaharlal Nehru University, New Delhi 110067, India; 3 Plant Stress Biology Group, International Centre for Genetic Engineering and Biotechnology, New Delhi 110067, India; 4 Plant Molecular Biology and Biotechnology Laboratory, Faculty of Veterinary and Agricultural Sciences, The University of Melbourne, Parkville, Melbourne, VIC 3010, Australia; 5 National Agri-Food Biotechnology Institute, Sahibzada Ajit Singh Nagar 140306, India

**Keywords:** abiotic stress, expression profiling, long-noncoding RNA, phytohormone, salinity

## Abstract

Pokkali is a strong representation of how stress-tolerant genotypes have evolved due to natural selection pressure. Numerous omics-based investigations have indicated different categories of stress-related genes and proteins, possibly contributing to salinity tolerance in this wild rice. However, a comprehensive study towards understanding the role of long-noncoding RNAs (lncRNAs) in the salinity response of Pokkali has not been done to date. We have identified salt-responsive lncRNAs from contrasting rice genotypes IR64 and Pokkali. A total of 63 and 81 salinity-responsive lncRNAs were differentially expressed in IR64 and Pokkali, respectively. Molecular characterization of lncRNAs and lncRNA-miRNA-mRNA interaction networks helps to explore the role of lncRNAs in the stress response. Functional annotation revealed that identified lncRNAs modulate various cellular processes, including transcriptional regulation, ion homeostasis, and secondary metabolite production. Additionally, lncRNAs were predicted to bind stress-responsive transcription factors, namely ERF, DOF, and WRKY. In addition to salinity, expression profiling was also performed under other abiotic stresses and phytohormone treatments. A positive modulation in TCONS_00035411, TCONS_00059828, and TCONS_00096512 under both abiotic stress and phytohormone treatments could be considered as being of potential interest for the further functional characterization of IncRNA. Thus, extensive analysis of lncRNAs under various treatments helps to delineate stress tolerance mechanisms and possible cross-talk.

## 1. Introduction

Rice is one of the most important staple crops widely consumed across the globe. The environmental challenges posed by different abiotic and biotic stresses can impair crop growth and development, ultimately leading to yield loss. Salinity, along with drought, is a major environmental stress that can lead to >50% yield losses in major crops worldwide [1]. Plants adapt to these harsh environmental conditions through various morpho-physiological, biochemical, and molecular responses to minimize the adverse effect of stress. Specifically, in-depth molecular investigations can play a significant role in developing superior genotypes for sustainable agriculture production. Recently, advancements in high-throughput sequencing technologies have made notable progress in decoding the transcriptional and post-transcriptional regulatory processes involved in the stress response. To date, numerous stress-responsive genes, transcription factors (TFs), non-coding RNAs, and stress-responsive proteins have been identified in rice and other crop plants. Along with genome mapping, advances in high-throughput sequencing have also helped in understanding a new category of RNA named long non-coding RNA (lncRNA).

The lncRNAs are non-coding transcripts with a length of >200 nt. Like other regulatory RNAs, these transcripts also coordinate biological processes across the plant and animal kingdoms. In plants, including rice, lncRNAs have been identified at different developmental stages, in specific tissues, or in response to biotic and abiotic stresses [2,3]. Like coding genes, lncRNAs are also transcribed by RNA polymerase II and act as a precursor to miRNAs and siRNAs [4]. Additionally, some Pol IV/V-dependent lncRNAs with an ability to control RNA-directed DNA methylation (RdDM) have also been Identified [5]. A study in rice that identified 2224 lncRNAs in pistils, anthers, shoots, and seeds revealed lncRNAs to be highly stage- and tissue-specific [6]. Recently, several studies have also focused on understanding the diverse functions of lncRNAs in response to environmental stresses. Chung et al. [7] identified 98 drought-responsive lncRNAs expressed in rice. Similarly, PLncPRO, a computational pipeline for predicting lncRNAs, detected 3714 high-confidence lncRNAs in different rice genotypes under drought conditions and salinity stress [8]. Interestingly, very few of these stress-responsive lncRNAs identified through RNA-seq data have been further validated. Work from our laboratory has focused on understanding the complex molecular machinery engaged in the salinity response in rice at the seedling stage. Previously, our laboratory proved the contrasting behavior of IR64 and Pokkali under salinity stress on the basis of variable phenotypes [9], as well as via decoding the complex cellular machinery at transcriptome [10] and proteome [9,11] levels [12]. It is known that Pokkali is taller in height and has ellipsoidal seeds; however, IR64 is semi-dwarf and has slender seeds.

The present study aimed to identify and characterize a repertoire of salinity-responsive lncRNAs in contrasting rice genotypes, and further explore the role of selected lncRNAs under multiple abiotic stress and phytohormone treatments to uncover potential cross-talk among the stress responses. Further analysis of this data would contribute new dimensions to prior knowledge of developing transgenic rice crops with improved stress tolerance, ultimately leading to an enhanced grain yield.

## 2. Results

### 2.1. Genome-Wide Identification of O. sativa lncRNAs

**Figure 1:**
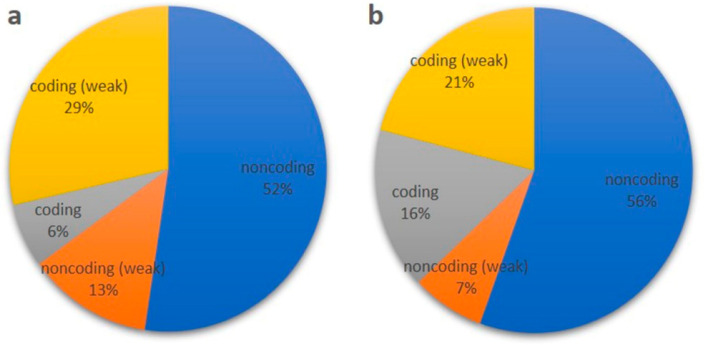
(**a**,**b**): Screening of coding potential of identified lncRNAs of rice genotype (**a**) IR64 and (**b**) Pokkali using a Coding Potential Calculator (CPC). The transcripts with scores between −1 and +1 are marked as ‘weak noncoding’ or ‘weak coding’, while transcripts with scores of <−1 and >1 are marked as ‘noncoding’ or ‘coding’.

The high-confidence set (probability cutoff of ≥ 0.8) of lncRNAs was identified in rice, based on the RNA-seq data of the rice genotypes IR64 and Pokkali in the previous study using PLncPRO [8]. Among the total lncRNAs, 63 and 81 were significantly differentially expressed in IR64 and Pokkali, respectively, under salinity stress, out of which 17 were shared in both of the genotypes. Details of the selected RNA-seq data of both the contrasting genotypes are summarized in Table S2. Furthermore, on the removal of a >0 CPC score, only 41 noncoding lncRNA in IR64 and 51 in Pokkali remained (Figure 1a,b; Table S2), with no ORFs detectable in these lncRNA. The remaining lncRNAs were either belonging to noncoding (weak) or coding (weak) category with small ORF (smORF). Furthermore, these potential noncoding transcripts were also subjected to a BLAST search against the RiceLncPedia database. After removing duplicates, only 18 lncRNAs in IR64 and 22 in Pokkali were obtained, as the remaining ones showed no hits (Table S3).

### 2.2. Characterization of the Identified lncRNAs

Further characterization of the predicted lncRNAs was done to understand their relevance in the rice genome. These characteristics were analyzed in both genotypes separately to gain insight into the variation in stress responses of two contrasting genotypes.

#### 2.2.1. Chromosomal Distribution

**Figure 2:**
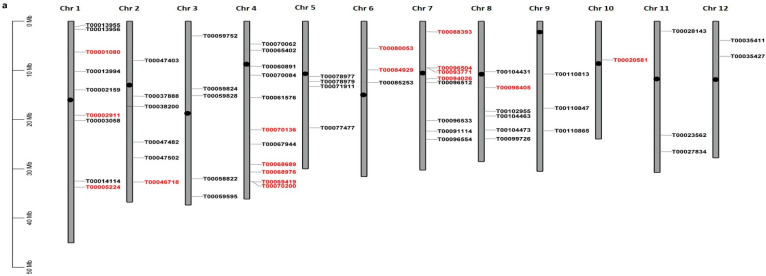

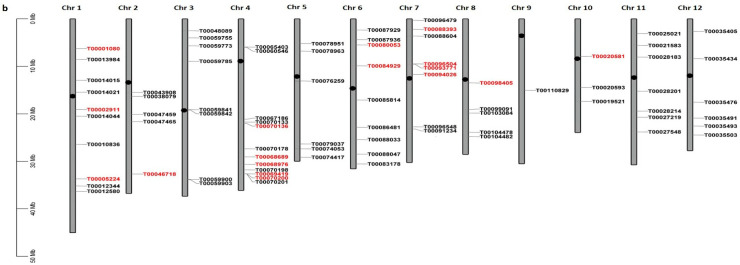
(**a**,**b**): A physical map of rice showing the location of lncRNA in rice genotypes (**a**) IR64 and (**b**) Pokkali. LncRNAs shared by both genotypes are highlighted in red. Black dots showed the position of centromere on chromosome.

The entire genome of *O. sativa* is divided into 12 chromosomes. The distribution of salinity-responsive lncRNAs across the 12 chromosomes in both genotypes was mapped (Figure 2a,b). The lncRNAs were found to be distributed unevenly, with the highest and lowest percentage of abundance on different chromosomes. Interestingly, the highest density of lncRNA genes was found on chr4 in both genotypes, whereas chr9 and chr10 contained the lowest number of lncRNAs in the IR64 and Pokkali genotypes. Furthermore, 32% and 45% of the lncRNAs were transcribed from the telomeric region of chromosomes in IR64 and Pokkali, respectively (Figure S1).

#### 2.2.2. Length Distribution and Subcellular Localization

**Figure 3:**
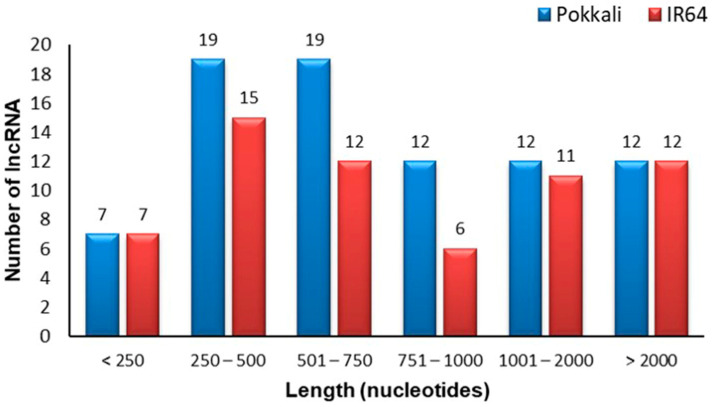
Length distribution of identified lncRNAs from rice genotypes IR64 and Pokkali.

**Figure 4:**
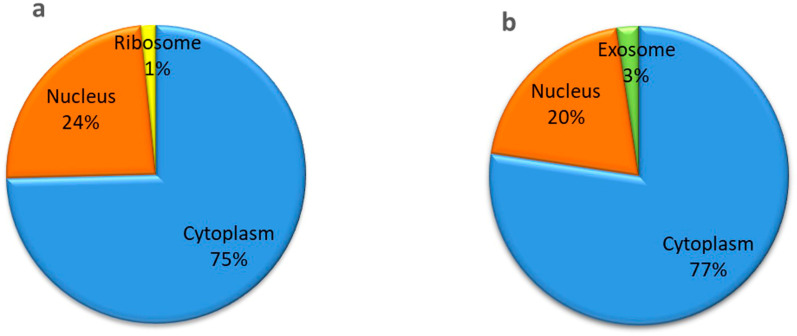
(**a**,**b**): Subcellular localization of lncRNAs in rice genotypes (**a**) IR64 and (**b**) Pokkali.

The length of the identified salinity-responsive lncRNAs in both genotypes ranged from approximately 200 to 5359 bp. However, among these, 43% of lncRNAs in IR64 and 47% in Pokkali ranged from 250 to 750 nucleotides (Figure 3; Table S4). While examining their location, it was found that most of the lncRNAs (~75%) mainly reside in the cytoplasm, with a smaller percentage located in the nucleus (~20%) in both genotypes (Figure 4a,b; Table S4). However, a few of the lncRNAs were localized in ribosome and exosome (Figure 4a,b; Table S4).

#### 2.2.3. Classification and Other Related Traits

The lncRNAs were classified based on their genomic locations and assigned to different categories (Table S3). Few lncRNAs were classified as ‘long noncoding isoforms of known genes’, which means this group of transcripts has at least one spliced site in common with the reference transcripts. Among these lncRNAs, some were reported to work as a precursor miRNA (pre-miRNA), or to contain a transposable element (Table S3). In IR64, TCONS_00047502, and TCONS_00096533 and in Pokkali, TCONS_00070178 were found to have transposable elements. Additionally, in Pokkali, TCONS_00087936 acted as pre-miRNA for osa-miR812b. A blast search against the RiceLncPedia database also showed some of the lncRNAs to be co-located with QTLs linked to traits specific to various tissues, development stages, or stress tolerance (Table S3). Specifically, TCONS_00027834 and TCONS_00068689 in IR64 and TCONS_00043908 and TCONS_00068689 in Pokkali showed localization with the QTL gene having a trait for salinity tolerance. Interestingly, the QTL gene qSDS-7 related to TCONS_00019521 lncRNA of Pokkali has a trait of salt sensitivity.

### 2.3. Expression Profile of the Identified O. sativa lncRNAs

To identify salinity-responsive long non-coding RNAs from the contrasting genotypes, FPKM values were obtained from the PLncPro database with FPKM > 1. Among the total lncRNAs, we identified 63 and 81 differentially expressed transcripts in IR64 and Pokkali, respectively (Table S2). Based on the log2 fold-change expression values compared to the control, the salt stress-responsive lncRNAs were divided into three categories: low (≥1 and <5), moderate (≥5 and <10), and high (>10) expression. The expression data revealed that differentially expressed lncRNAs mostly reside in the low and moderate expression categories (Table S2).

### 2.4. Target Identification of the lncRNAs in O. sativa

To gain a comprehensive view of the possible lncRNA-miRNA interactions, salt-responsive lncRNAs identified from both the genotypes were subjected to analysis with the psRNATarget tool to identify their target miRNAs. We determined that only 50 salt-responsive lncRNAs from IR64 and 63 from Pokkali target 311 and 364 miRNAs, respectively (Table S5). Well-known salt-responsive miRNAs, including miR156, miR159, miR167, and miR5505, were also targeted by these lncRNAs. Targeted miRNAs1 obtained from both genotypes were further analyzed to find their target genes and study the interrelatedness between the lncRNA-miRNA and protein-coding genes. It was observed that a total of 1903 and 2176 genes were targeted by miRNAs of IR64 and Pokkali, respectively (Table S5). Interestingly, most lncRNAs regulate SBP domain-containing proteins, various TFs, retrotransposons, and several other expressed and hypothetical proteins.

### 2.5. Functional Annotation of the Salt Stress-Responsive lncRNAs in O. sativa

**Figure 5:**
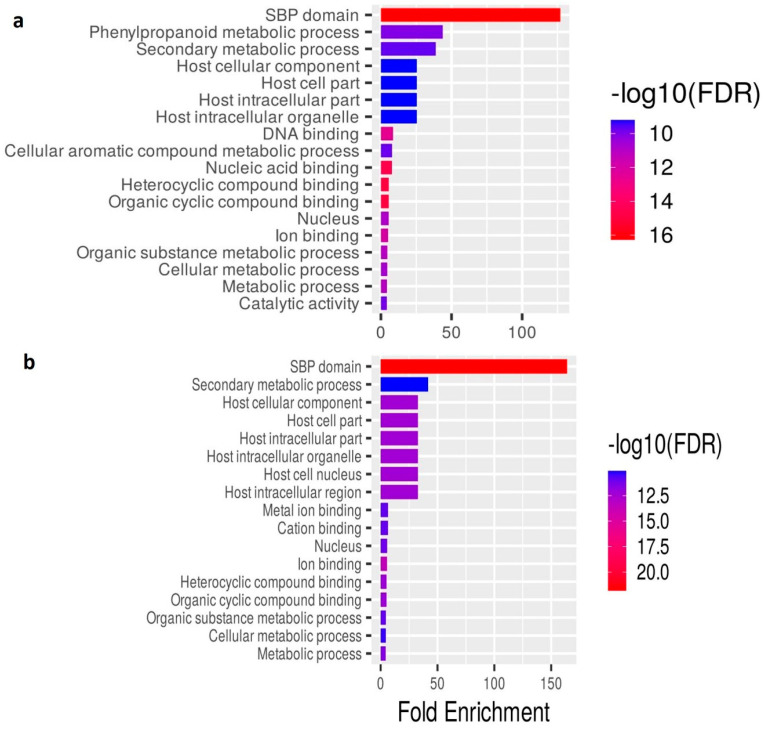
(**a**,**b**): Fold enrichment showing the percentage of genes belonging to a particular pathway in rice genotypes (**a**) IR64 and (**b**) Pokkali. Pathways have been filtered based on the FDR cut-off. The top 20 most significant pathways are shown here.

**Figure 6:**
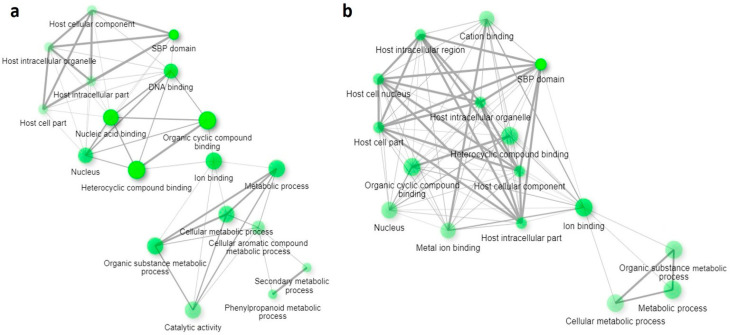
(**a**,**b**): Interactive plot showing the relationship between obtained gene ontology (GO) enriched pathways in (**a**) IR64 and (**b**) Pokkali, developed using ShinyGO 0.76 software. The pathways (nodes) are connected if sharing 20% (default) or more genes. Darker nodes show more significantly enriched gene sets. Bigger nodes represent larger gene sets. Thicker edges represent more overlapped genes.

To decipher the possible functions of the identified salt stress-responsive lncRNAs from both the genotypes, GO enrichment analysis was conducted, and the function-based annotation showed their involvement in the diverse role of ‘biological process’, ‘cellular components’, and ‘molecular function’ categories, mainly related to the transcriptional gene regulation and development process (Table S6). It was observed that GO terms related to ion transport and homeostasis in the biological process are comparatively higher in Pokkali than in IR64. Pokkali GO terms related to ion homeostasis are GO:0000041, GO:0030001, GO:0055065, GO:0055072, and GO:0006826. Apart from this, the lignin metabolic process and plant organ development were also abundantly found in both genotypes. While, in the case of cellular components, intracellular organelle (GO:0043227, GO:0043229), cellular anatomical entity (GO:0110165), and transcriptional regulation (GO:0090575) featured prominently in both genotypes. The molecular function category primarily includes binding (GO:0005488) GO terms in both genotypes. Interestingly, ion binding (GO:0043167) was specifically found in Pokkali. In fold enrichment analysis, the SBP domain exhibits maximum frequency in both genotypes (Figure 5a,b). However, the phenylpropanoid metabolic process was found to be exclusively dominant in IR64. Additionally, it was observed that lncRNA functioning was related to the nuclear processes or ion binding. Furthermore, the interactive network was plotted to analyze the relationship between the obtained enriched pathways (Figure 6a,b). Hierarchical clustering of significantly enriched pathways showed the correlation among the enriched pathway. Pathways with many shared genes were clustered together. According to the clustering data in IR64, nucleic acid binding, DNA binding, cyclic compound binding, and SBP domain were significantly prominent. However, in Pokkali, the SBP domain, ion binding, and metabolic processes were the most prominent ones (Figure S2a,b).

### 2.6. Association of Salt-Responsive lncRNAs with Transcription Factors in O. sativa

**Figure 7:**
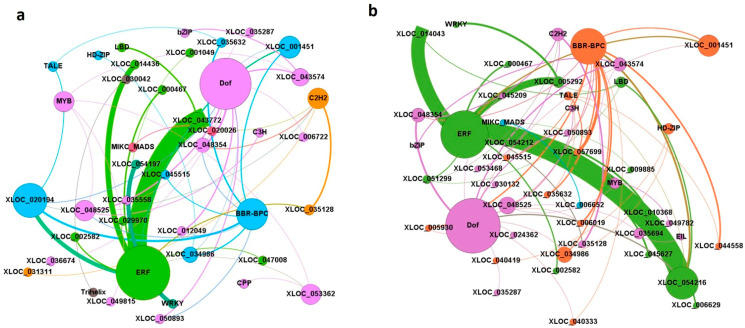
(**a**,**b**): Interaction network showing the association of lncRNAs with transcription factors obtained from the plant transcription factor database v4.0 (PlantTFDB), developed using Gephi 0.9.1 software in (**a**) IR64 and (**b**) Pokkali. Nodes represent transcription factors and edges represent lncRNA. Node size is proportional to the number of interactions. Thick edges show that interaction occurred together more often than in those with a thinner edge. Different colors have been used to differentiate components from each other.

Based on the sequence information available for *O. sativa* in the Plant TF database (PlantTFDB) v4.0., binding sites for *O. sativa* lncRNAs were identified. It was observed that 28 and 34 lncRNAs from IR64 and Pokkali, respectively, bind with 13 TF families indicating their potential involvement in response to salt (Table S7). The highest percentage of co-expressing lncRNA-TF pairs was observed for ERF, followed by BBR/BPC, DOF, and WRKY in both of the genotypes (Figure 7a,b).

### 2.7. Expression Analysis of lncRNA Candidates under Salinity Stress

**Figure 8:**
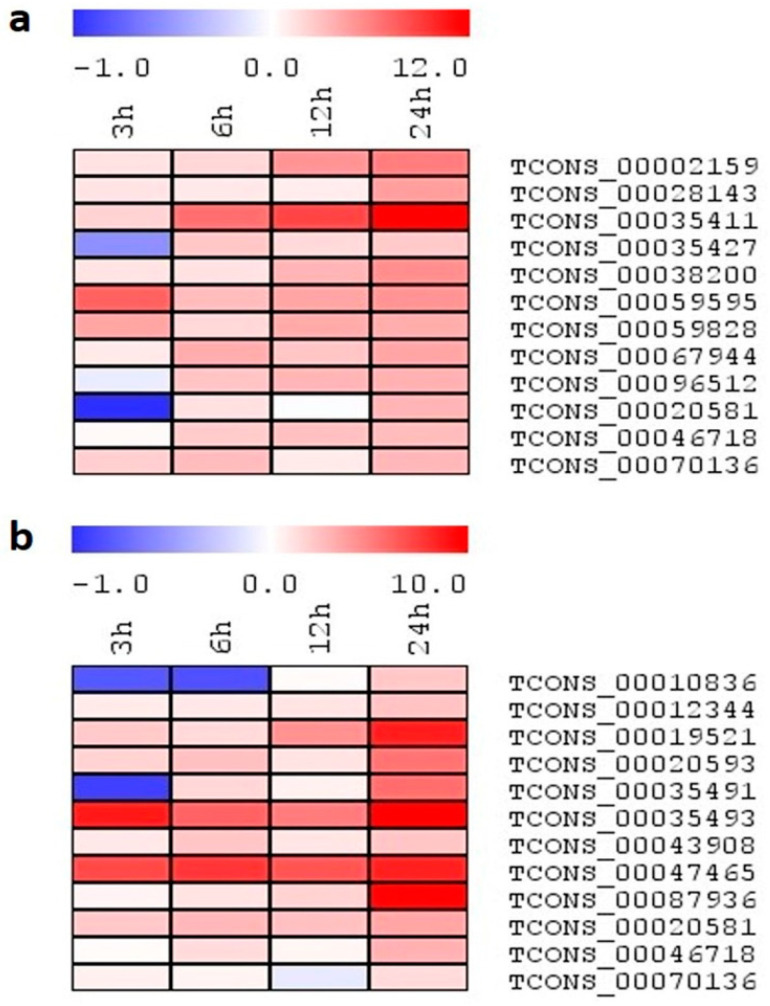
(**a**,**b**): The heatmap generated based on the log2 fold-change values showing differential expression of lncRNAs in rice genotypes (**a**) IR64 and (**b**) Pokkali exposed to salinity at 0, 3, 6, 12, and 24 h. The color scale for fold-change values is shown at the top.

Expression analysis using the qRT-PCR platform helps provide further information regarding the function of transcripts, and also helps validate the RNA-seq-based expression values. Therefore, to analyze the lncRNAs regulation in rice, the expression profiles of selected lncRNAs were investigated in two-week old rice seedlings subjected to salinity stress (Figure 8a,b). Apart from 24 h of stress treatment, we also studied the expression profiling of lncRNAs under salinity stress for early time points, viz., 3 h, 6 h, and 12 h. A total of 20 lncRNA transcripts were randomly selected to validate the expression in both genotypes. From these, five transcripts were common between the contrasting genotypes, and 15 were unique, belonging to their respective genotypes. From these selected lncRNAs, primers of a few transcripts failed to amplify expected transcripts. However, most of the transcripts were validated by qRT-PCR and showed enhanced expression following all stress durations, especially at 24 h time points in both the genotypes. In Pokkali, TCONS_00019521 and TCONS_00047465 showed a progressive expression during stress duration, with a maximum value up to ~8-fold. The expression of TCONS_00035493 induced at early stress (3 h) remained constitutive until 24 h, with a maximum value of ~10-fold. In TCONS_00087936, basal expression was observed until 12 h of stress, after which point an abrupt ~10-fold increase was noted at 24 h. Similarly, in IR64, TCONS_00035411 showed an increased expression of lncRNA, with the progression of stress with a maximum value of ~13-fold at 24 h. In contrast to other transcripts, Only TCONS_00059595 showed maximum induction at 3 h, after which point, expression slightly reduced with the progression of stress duration. In addition to the unique transcripts, shared ones also showed enhanced expression under stress, but the pattern of their induction differed between the genotypes. As in Pokkali, TCONS_00020581 showed induction in expression by ~3-fold at all time points, while in IR64, induction was observed at 24 h only (~3.5-fold). In the case of TCONS_00046718, a contrasting expression was observed, Pokkali showed induction (~3-fold) at 24 h only, while IR64 showed enhanced expression at all time points (~3-fold). TCONS_00070136 showed basal expression in Pokkali, while in IR64, its expression gets induced at 3 h and progressively increases up to ~3-fold until 24 h.

### 2.8. Expression Profiling of Select lncRNA Candidates under Different Abiotic Stresses

**Figure 9:**
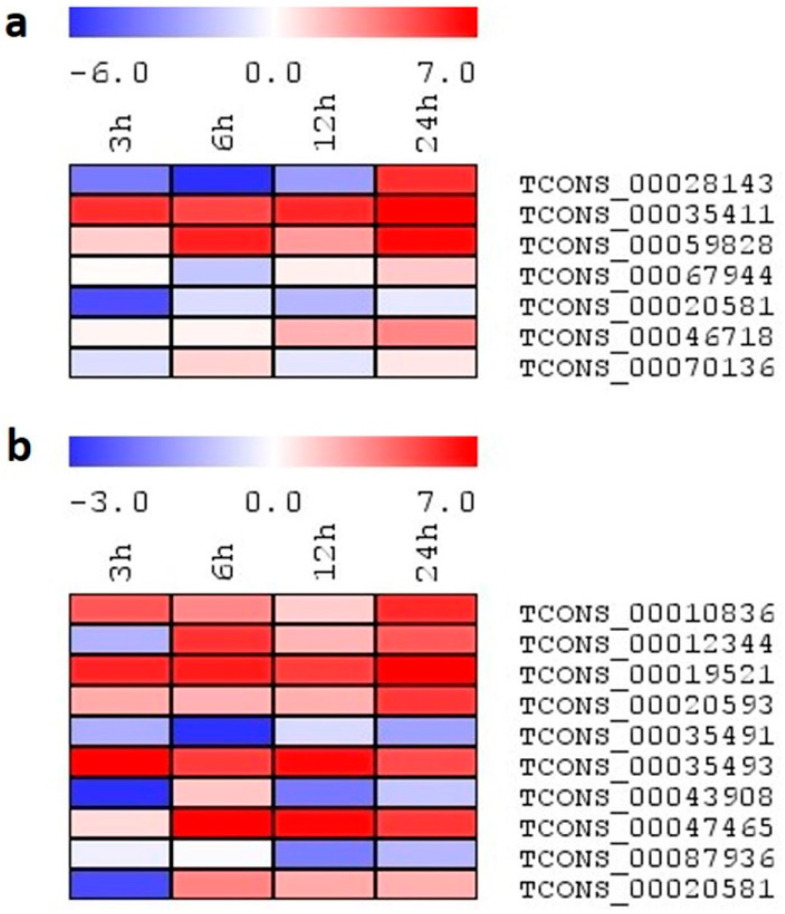
(**a**,**b**): The heatmap generated based on the log2 fold-change values showing differential expression of lncRNAs in rice genotypes (**a**) IR64 and (**b**) Pokkali exposed to osmotic stress at 0, 3, 6, 12, and 24 h. The color scale for fold-change values is shown at the top.

Based on the expression profile of lncRNAs in rice seedlings under salinity, salt-induced lncRNAs with higher expression values were further selected for detailed expression analysis under different abiotic stresses (osmotic stress, heat, cold) at similar stress durations. Under osmotic stress, 10 and 7 transcripts showed expression in IR64 and Pokkali, respectively (Figure 9a,b). In Pokkali, all transcripts generally showed upregulation upon PEG treatment, except TCONS_00035491 and TCONS_00043908. TCONS_00035491 showed a downregulation by ~3-fold at 6 h, after which point, expression tends towards the basal level. Similarly, TCONS_00043908 showed a downregulation at 3 h by ~3-fold after that basal transcript level was observed. TCONS_00010836, TCONS_00019521, and TCONS_00020593 showed higher transcript accumulation at all time points, with expression levels ranging between 5- and 7-fold at 24 h. Only TCONS_00047465 showed a progressive increment in the transcript level with the progression of stress duration. In IR64, TCONS_00035411 and TCONS_00059828 showed induced expression at all time points, with a higher value of ~7-fold at 24 h. Enhanced expression was also observed from 6 h to 24 h in TCONS_00059828, with a fold change value of ~6-fold. TCONS_00028143 showed a downregulation until 12 h; however, an abrupt incremental increase in the transcript was observed at 24 h by ~6-fold.

**Figure 10:**
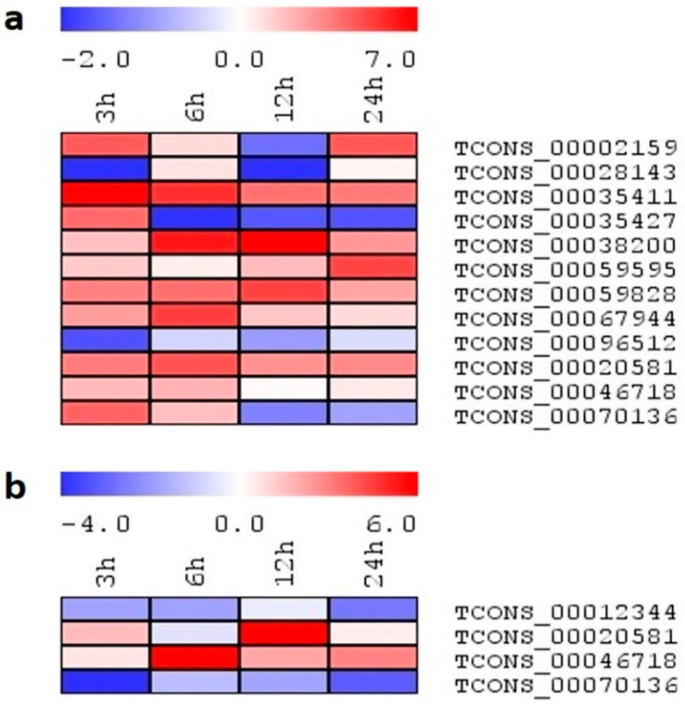
(**a**,**b**): The heatmap generated based on the log2 fold-change values showing differential expression of lncRNAs in rice genotypes (**a**) IR64 and (**b**) Pokkali exposed to heat at 0, 3, 6, 12, and 24 h. The color scale for fold-change values is shown at the top.

Furthermore, to study the role of lncRNAs under temperature stress, both genotypes were exposed to high and low-temperature stress. Under heat stress, maximum transcript expression was observed in IR64 (12), while only four transcripts were expressed in Pokkali (Figure 10a,b). Out of the four lncRNAs, only TCONS_00046718 showed increased expression from 6 h to 24 h and TCONS_00020581 enhanced expression by ~6-fold at 12 h. In the remaining two lncRNAs, no stimulation was observed in TCONS_00012344, while TCONS_00070136 showed less transcript accumulation. Similar to Pokkali, TCONS_00020581 showed enhanced expression by ~3-fold at all time points in IR64. TCONS_00070136 showed an upregulation by ~4-fold after 3 h of heat exposure; however, its expression decreased with the progression of stress duration. TCONS_00035411, TCONS_00038200, and TCONS_00067944 showed enhanced expression at all time points, with a maximum value of ~7-fold at 3 h, 12 h, and 6 h, respectively. Additionally, a ~4-fold expression was observed in TCONS_00035427 at 3 h, and a ~5-fold increment was noted in TCONS_00059595 at 24 h, while other time points showed basal expression.

**Figure 11:**
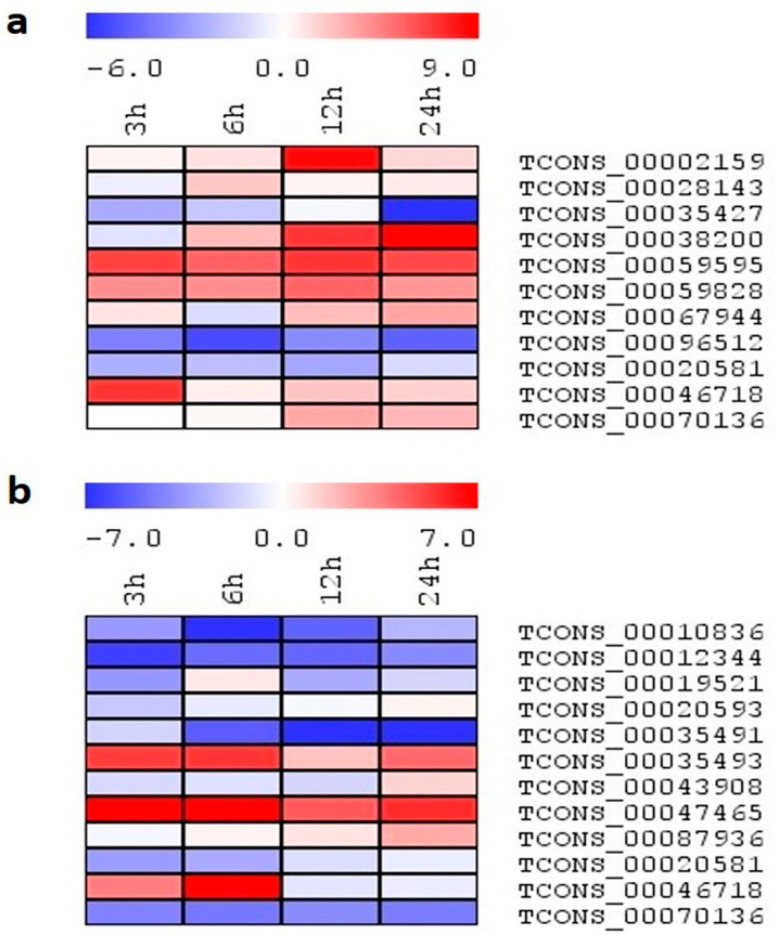
(**a**,**b**): The heatmap generated based on the log2 fold-change values showing differential expression of lncRNAs in rice genotypes (**a**) IR64 and (**b**) Pokkali exposed to cold at 0, 3, 6, 12, and 24 h. The color scale for fold-change values is shown at the top.

When plants were subjected to low temperature, most of the lncRNAs showed either a downregulation or basal expression in Pokkali (Figure 11a,b). Higher transcripts were accumulated at all time points in TCONS_00035493 and TCONS_00047465, and a progression of stress decline in the fold change was observed. However, TCONS_00046718 showed increased expression at 3 h and 6 h only. In the case of IR64, TCONS_00035427, TCONS_00096512, and TCONS_00020581 showed a downregulation under low temperature treatment. However, TCONS_00038200, TCONS_00059595, and TCONS_00059828 showed upregulation at all stress durations, with a maximum value of ~10-fold at 24 h and ~7-fold and ~5-fold at 12 h, respectively. Moreover, TCONS_00002159 showed an upregulation by ~8-fold only at 12 h, and TCONS_00067944 showed a slight induction by ~3-fold only at 24 h of low temperature treatment.

### 2.9. Expression Analysis of Selected lncRNAs on Exogenous Application of Phytohormones

It is well known that stress signaling in plants is interconnected with signaling machinery engaged in plant growth regulators. Hence, we also studied the likely role of selected lncRNAs for phytohormonal regulation by analyzing the expression of lncRNAs under phytohormone treatments—such as ABA, SA, JA, and ET—in the contrasting genotypes. In Pokkali, upon exogenous application of ABA, only TCONS_00035493 showed an upregulation by ~9-fold at all durations (Figure S3). However, TCONS_00012344 was downregulated by ~2-fold at all time points. The remaining transcripts were either expressed at the basal level or not expressed under ABA treatment. In the case of IR64, only TCONS_00059828 showed induced expression at 3 h and 12 h by ~4-fold, while TCONS_00096512 and TCONS_00020581 were found to be downregulated at all time points (Figure S3). Additionally, when Pokkali was treated with SA, no transcript was found to be upregulated. However, TCONS_00012344 and TCONS_00020581 showed a downregulation in their expression by ~4-fold at the later time points, i.e., 12 h and 24 h. In IR64, TCONS_00059828 showed enhanced expression by ~4-fold at all time points, and TCONS_00067944 showed an upregulation by ~3-fold at 3 h and 12 h only (Figure S4). However, TCONS_00096512 was highly downregulated at all durations under SA treatment. On application of JA, TCONS_00012344, TCONS_00020581, and TCONS_00070136 were downregulated at all time points in Pokkali, while in IR64, the expression of TCONS_00096512 and TCONS_00020581 progressively declined with treatment. However, TCONS_00047465 showed an upregulation by ~3-fold in Pokkali, and TCONS_00059828 showed induction by ~4-fold in IR64 at all time points (Figure S5). Furthermore, it was observed that the exogenous treatment of ET induced the TCONS_00047465 transcript in Pokkali by ~5-fold and ~8-fold at 3 h and 24 h, respectively. In contrast, TCONS_00020581 was upregulated by ~2-fold at 24 h only. However, no transcript was found to be upregulated in IR64. In Pokkali, TCONS_00012344 and TCONS_00070136 showed a downregulation by ~5-fold and ~2-fold at all time points of the treatment. In contrast to Pokkali, TCONS_00020581 in IR64 showed a downregulation upon ET treatment (Figure S6).

## 3. Discussion

Several studies have been undertaken in recent years to identify and characterize lncRNAs in various plant species. Several computational studies have now been conducted for lncRNA identification in rice, but a big gap remains in their validation and functional characterization. Few studies have focused on their role under stress conditions, particularly abiotic stresses [13,14]. Environmental challenges impede the growth and development of crop plants, leading to yield decline in crop plants. Therefore, it is imperative to understand the molecular basis of plant stress tolerance mechanisms, specifically involving lncRNAs, to identify candidate transcripts that can be deployed to develop improved stress-tolerant crop genotypes. In the present study, we identified and characterized the salt stress-responsive lncRNAs in both rice genotypes, i.e., IR64 and Pokkali, using computational approaches, and performed expression analysis to investigate the temporal effects of stress under various short-term abiotic stresses and phytohormone treatments (Figure S7). To our knowledge, this is the first study that examines the multifaceted role of lncRNAs in seedlings of contrasting rice genotypes in response to abiotic stresses and phytohormone treatments to delineate the mechanism of a possible cross-talk among them. Being a tolerant genotype, a comparatively higher number of salt-responsive lncRNAs were identified in Pokkali than IR64, indicating the role of these non-coding RNAs in complex salinity stress-responsive regulatory networks. Variance in the lncRNA coding potential, i.e., weak noncoding and weak coding category, it is evident that lncRNA have the ability to code for short peptides [15]. Additionally, the presence of small ORF (smORF) also stipulates that lncRNA may be translated in short functional peptides, and could also be the reason for the activation of nonsense-mediated decay pathways [15]. A subcellular localization study inferred the vast functionality of lncRNAs within the cells. LncRNA present in the nucleus significantly participates in epigenetically regulated gene expression, such as enhancing the chromatin loop and regulating chromatin status, as well as playing a role in silencing, splicing, etc. [16]. Additionally, cytoplasmic lncRNAs significantly associate with RNA-binding proteins (RBPs) to regulate the translation of mRNAs, activate signaling molecules, and facilitate protein degradation machinery [17]. In our study, most of the lncRNAs were found to be localized in the nucleus as well as cytoplasm, indicating an lncRNA-mediated stress tolerance mechanism in rice. Moreover, a few lncRNAs were also found to reside in the ribosome and exosome. The presence of lncRNAs in ribosome supports the translation of lncRNA into small peptides; however, the actual mechanism is unknown [18]. The existence of lncRNA in exosome indicates the mediation of intercellular communication by carrying lncRNAs. Exosome-containing lncRNAs can be secreted and end up in recipient cells to become involved in epigenetic control, cell-type re-programing, and genomic instability [18].

Transcription of some lncRNAs from the telomeric regions indicates their role in delaying cellular senescence and sustaining genome instability and epigenetic regulation. A comparatively higher number of lncRNAs residing in the telomeric region signifies delayed senescence and higher tolerance in Pokkali towards salinity stress. Both mammalian and plant telomeres are transcribed to long non-coding RNA called TERRA. This transcriptional potency may be due to the relatively lower compactness of telomeric chromatin than heterochromatin [19]. In Arabidopsis, a specific or maybe dominant fraction of TERRA is transcribed from interstitial telomeric sequences, which are purely heterochromatic [20,21]. The vast variance in length of lncRNAs also indicates the functional versatility within the cell, as recent reports have shown that lncRNAs have the potential to encode micropeptides that have the potential role in the growth and development of plants under normal, as well as stressed conditions [22]. The length of lncRNAs determines the arrest of epigenetic complex and, upon transcriptional termination, unmask 3′-degradation signals to limit the RNAs’ half-life and prevent diffusion at ectopic locations [23]. LncRNA also works as an endogenous target mimic to regulate gene expression by competing with miRNA. Hence, it can be said that the larger the lncRNAs, the higher the availability of binding sites for miRNA, i.e., there is a higher probability for miRNA sponging to sequester miRNAs from their target [24]. Variability in the functioning of lncRNAs is also evident from their classification as intergenic/intronic, sense/antisense, and cis/trans. The presence of transposable elements in a few lncRNAs in both genotypes also supports their role in epigenetic regulation [25]. As stated above, lncRNA functions as an miRNA sponge. Hence, we intended to study lncRNA-miRNA interaction. Interestingly, it was observed that miR156, miR159, miR167, and miR5505, which are well known for their involvement in salinity tolerance, were also found to be regulated by lncRNA in rice genotypes [26,27,28]. Fascinatingly, various miRNAs found in Pokkali have interaction with lncRNA, but these interactions were absent in the case of IR64 under salinity. Some of miRNAs that specifically showed interaction with lncRNA in Pokkali under salinity were miR1427, miR164, and miR167, indicating a higher tolerance capability [29]. Additionally, the QTL gene qSDS-7, specifically found in Pokkali, has a trait of salt sensitivity, which is also evident in the Gramene QTL database. All these obtained salinity responsive QTL are the QTLs that are apart from *Saltol* QTL. However, based on the genomic location, some lncRNAs have start and end positions within *Saltol* QTL regions, and can hence be considered to be co-localized with *Saltol* QTL. Furthermore, to establish interrelatedness among lncRNA-miRNA-mRNA, selected mRNAs were further subjected to GO enrichment analysis to evaluate the functioning of lncRNA in biological pathways. A fold enrichment study showed that in both genotypes, the maximum percentage of genes belong to the ‘SBP domain’ (Squamosa-Promoter Binding Protein), with the highest percentage in Pokkali. This domain is a sequence-specific binding domain of plant protein, and is well known for its involvement in salinity and drought tolerance in plants, including rice [30,31]. Subsequently, a significant presence of ‘phenylpropanoid metabolic pathway’ in IR64 evident the generation of an enormous array of secondary metabolites—such as lignin, flavonoid, and coumarin—to scavenge harmful ROS produced under stress [32]. It might be possible that this category was not discerned in Pokkali due to its high salt-tolerant nature. Our analysis of the fold enrichment study revealed that most enriched terms corroborate the stress tolerance mechanism, with the DNA binding domain as the utmost priority.

As lncRNAs are predominantly involved in gene regulation, we investigated their putative interaction with transcription factors (TFs), to see if the two regulatory moieties function in synergy in response to abiotic stress. ERF, MYB, bZIP, and WRKY are known for their contribution to stress tolerance [33]; their association with lncRNA showed their involvement in TFs-driven stress tolerance. A recently-published review by Tiwari et al. [34] also reported the involvement of these TFs in salinity tolerance, particularly in the contrasting rice genotypes used in the present study.

The study of transcript abundance also suggested a differential expression profile of the identified salinity-responsive rice lncRNAs. RNA-Seq data analysis showed salt-responsive lncRNAs exhibit up and downregulation expression patterns. However, most of the lncRNAs were low or moderately expressed compared to protein-coding transcripts. For the validation of RNA-Seq values, expression profiling was done using qRT-PCR at both early and late time points of stress. It was observed that most of the selected lncRNAs were upregulated, indicating their induction upon salinity stress. In a study, lncRNA Osa01LNT0019900.1 and Osa01LNT0023300.1 showed a higher similarity to TCONS_00068689 and TCONS_00043908 of Pokkali, respectively. They also showed enhanced expression under salinity stress in Baldo (Indica) and Vialone nano (Japonica) rice [35]. To study the multi-level regulation by lncRNAs for abiotic stress tolerance, we further analyzed the expression of highly upregulated salinity-responsive lncRNAs under various abiotic stresses, viz., osmotic stress, heat and cold, and observed that selected salt-responsive lncRNA shared an expression profile under these stress treatments as well. Most of the salt-responsive lncRNAs were upregulated in osmotic stress; however, very few overlaps were observed in the case of heat and cold stress. Variance observed in the expression patterns of selected transcripts suggested the multifaceted role of these lncRNAs in regulating the pathways involved in stress tolerance and adaptation under the influence of multiple abiotic stresses. As phytohormones are well-documented as playing a crucial role in stress tolerance, we exogenously applied ABA, SA, JA, and ET to study the specific response of the particular lncRNA. However, very few overlaps of salt-responsive lncRNAs were observed with phytohormone treatments. Among the selected lncRNAs, only TCONS_00059828 of IR64 showed expression under all the phytohormone treatments. In the case of Pokkali, TCONS_00047465 showed comparatively higher expression on the exogenous application of JA and ET. A recent study reported that exogenous application of JA alleviates salt stress by increasing the antioxidant response in plants [36]. Studies also proposed that ethylene significantly regulates salinity stress responses by maintaining Na^+^/K^+^ homeostasis, and reactive oxygen species by triggering antioxidant defense [37,38]. A study by Lim et al. [39] showed interconnection between JA, ET, and ERF transcription factors in the regulatory function of leaf senescence under abiotic stress, including salinity. Earlier studies also reported the alteration in gene expression under short-term abiotic stress and exogenous phytohormone treatment, and established the interrelation between them [40,41]. However, a similar study with lncRNAs has not been performed to date. Modulation observed in the expression after phytohormonal application also indicates the multilevel and interconnected regulation by lncRNAs for stress tolerance.

## 4. Materials and Methods

### 4.1. Identification of lncRNAs in O. sativa Contrasting Genotypes

The high-confidence set (probability cutoff of ≥0.8) of lncRNAs identified in rice was based on the RNA-seq data of the rice genotypes IR64 (salt sensitive) and Pokkali (salt tolerant) from the available online database PLncPRO (ccbb.jnu.ac.in/plncpro (accessed on 28 April 2021)) [8]. Furthermore, the salt-responsive lncRNAs from contrasting genotypes showing significant differential expression (*p* ≤ 0.05 and log2 fold change values ≥ ±1) were obtained for analysis. For verification of obtained lncRNAs, BLAST analysis was also performed to compare our results with another reported *O. sativa* lncRNAs database named RiceLncPedia (http://3dgenome.hzau.edu.cn/RiceLncPedia#/ (accessed on 4 June 2021)).

### 4.2. Computational Approach for Characterization of Identified O. sativa lncRNAs

The coding potential of the obtained lncRNAs was rechecked using a coding potential calculator (CPC; http://cpc.cbi.pku.edu.cn (accessed on 25 May 2021)), following the standard procedure [42]. The open reading frames (ORFs) of the filtered transcripts were analyzed using the ORF Predictor (http://proteomics.ysu.edu/tools/OrfPredictor.html (accessed on 25 May 2021)). The subcellular localization of lncRNAs was predicted using Locate R (locate-r.azurewebsites.net (accessed on 6 July 2021)). The MapChart was used for the graphical representation of the lncRNAs on the chromosome and their presence in the telomeric region. The RiceLncPedia database was also accessed to predict lncRNA as a miRNA precursor, lncRNA from transposons, its collocation with QTLs, and its expression across various tissues, developmental stages, and abiotic stresses.

### 4.3. Association of the Identified lncRNAs with Transcription Factors (TFs)

To examine the cellular transcriptional regulation by lncRNAs, an association of TFs with the identified salt stress-responsive lncRNAs was investigated. Plant transcription factor database v4.0 (PlantTFDB) was used to identify the TF binding site with threshold *p*-value ≤ 1 × 10^−7^ (http://plantregmap.gao-lab.org/binding_site_prediction.php (accessed on 17 September 2021)). The interaction network of TFs and lncRNAs was developed using software Gephi 0.9.1 (https://gephi.org/) [43].

### 4.4. Interaction of lncRNAs with miRNAs and mRNAs

The interaction between miRNAs and the identified salt stress-responsive lncRNAs obtained from both rice genotypes was examined. Target sites for miRNAs in their respective lncRNAs were predicted using a plant small RNA target analysis server (psRNATarget), with the default parameters and maximum expectation value up to 5 (https://www.zhaolab.org/psRNATarget/ (accessed on 17 March 2022)). Further obtained miRNA were submitted to a server to analyze their interaction with mRNA using a maximum expectation value of 2.0.

### 4.5. Functional Annotation of the Identified lncRNAs

Functional annotation was carried out on the obtained miRNA-targeted genes. Gene ontology (GO) enrichment analysis was executed using ShinyGO 0.76 software (http://bioinformatics.sdstate.edu/go/ (accessed on 17 March 2022)) [44]. All query gene IDs were searched against *Oryza sativa* STRING-db, with an FDR cut-off value of 0.05. Fold enrichment was analyzed to determine how drastically genes of a certain pathway are overrepresented. The top 20 (default) most significant pathways were considered based on the cut-off value. A hierarchical clustering tree was constructed to study the correlation among obtained significant pathways. The interactive plot was executed to show the relationship between the enriched pathways.

### 4.6. qRT-PCR-Based Expression Analysis of lncRNAs

#### 4.6.1. Plant Materials

The expression of selected salt stress-responsive lncRNAs was examined in the contrasting genotypes of *Oryza sativa* named IR64 (salt-sensitive) and Pokkali (salt-tolerant), obtained from our laboratory at School of Life Sciences, Jawaharlal Nehru University, New Delhi. The experiment was carried out in the growth chamber with three replications under the following conditions: temperature 28 ± 1 °C, photoperiodic cycle 16/8 h (light/dark), 50% relative humidity with a photon flux density of 700 μmol photons m^−2^ s^−1^. Hydroponically grown two-week-old rice seedlings of both genotypes were subjected to 200 mM NaCl for salt stress in Yoshida medium for 3, 6, 12, and 24 h. Furthermore, the expression profiles of selected lncRNAs for various abiotic stresses and phytohormone treatments were examined by exposing two-week-old rice seedlings of both genotypes to osmotic stress (20% PEG 6000), Heat (42/29 °C day/night temperature), cold (4 °C), 100 μM abscisic acid (ABA), 100 μM salicylic acid (SA), 100 μM methyl jasmonate (JA), and 100 μM ethephone (ET) for similar durations. Unstressed seedlings grown in plain Yoshida medium were considered the control. Harvested tissues were snap-frozen in liquid nitrogen and kept at −80 °C for qRT-PCR analyses.

#### 4.6.2. RNA Extraction and qRT-PCR Analysis

Total RNA was isolated from the rice seedlings by Spectrum™ Plant Total RNA Kit (Sigma, St. Louis, MO, USA). The RNA purity was interpreted by absorption ratio OD_260_/OD_280_ (1.9–2.0) and OD_260_/OD_230_ (≥2.0) on nanodrop (Thermo Scientific, Waltham, MA, USA). The cDNA synthesis was done using DNase-free total RNA primed with random hexamer primers by Maxima H Minus first-strand cDNA synthesis kit (Thermo Scientific, USA). Quantitative real-time PCR (qRT-PCR) was performed using PowerUp™ SYBR™ Green Master Mix (Applied Biosystem, Waltham, MA, USA) on CFX96 Real-Time PCR Machine (Biorad, Hercules, CA, USA) using *Ubiquitin* as the reference gene, as described elsewhere [45]. The list of primers used in the study is given in Table S1. The total abundance of transcripts was examined using 2^−ΔΔCt^ method [46]. The expression profiling data were visualized by heat map using the TIGR MultiExperiment Viewer (MeV 4) version 4.0.9 software package [47].

## 5. Conclusions

A thorough analysis of transcriptome and proteome data of contrasting genotypes IR64 and Pokkali, previously done in our laboratory, indicated that distinct stress-related genes and protein categories are expressed differently, which helps to explain the higher level of stress tolerance of Pokkali, which is a traditional salt-tolerant landrace. However, not much is known about how these genotypes respond differently in the context to lncRNA. Hence, for the comprehensive study of lncRNA-regulated salinity tolerance, we chose contrasting genotypes for our investigations. Rice cultivation entails transferring young seedlings to the field in order to preserve their capacity for tillering and rooting. As a result, this seedling stage is thought to be crucial for determining whether or not crop production will be successful. Hence, in the present study, we used the seedling stage to unravel the salinity tolerance mechanism mediated by lncRNA. The study showed that Pokkali has a comparatively higher number of salinity-responsive lncRNAs, which indicates it has evolved a more complicated mechanism for salinity tolerance over time. It is also evident from the in silico analysis that showed a higher involvement of Pokkali lncRNAs with various biochemical and molecular pathways than IR64, indicating that Pokkali has a comparatively more complex regulatory network for stress tolerance than IR64. Data from expression profiling at the early and late time points in response to one or more environmental stress stimuli could help to identify putative candidate lncRNAs playing a key role in stress tolerance. Their subsequent functional validation will contribute to advancing stress resistance crop improvement initiatives. Since TCONS_00002159, TCONS_00035411, TCONS_00059828, and TCONS_00096512 demonstrated positive modulation during both abiotic stress and phytohormone treatments, these may be considered a potential lncRNA of interest for further in vitro and in vivo functional characterization. Conclusively, specific lncRNAs in rice have temporal and stimuli-specific effects and provide a deeper understanding of the molecular mechanism underlying lncRNA-mediated stress tolerance and adaptation in plants, and help in developing stress tolerant crops in future.

Moreover, it would be fascinating to revisit the regulatory networks linked with lncRNAs, miRNAs, and TFs to identify breeding possibilities for improving stress tolerance in essential crop plants. Therefore, our findings have laid the foundation for the functional characterization of possible lncRNAs to decipher their specialized roles, as well as to set a new paradigm for understanding complex stress tolerance mechanisms in crop plants.

## Data Availability

No new data were created in this study. Data sharing is not applicable to this article.

## Supplementary Materials

The following supporting information can be downloaded at: https://www.mdpi.com/article/10.3390/ijms241411677/s1.

## References

[1] Bray E.A., Bailey-Serres J., Weretilnyk E., Buchanan B.B., Gruissem W., Jones R.L. (2000). Responses to abiotic stresses. Biochemistry and Molecular Biology of Plants.

[2] Tiwari S., Lata C., Upadhyay S.K. (2021). Recent advancements in long noncoding RNA-mediated stress responses in rice. Long Noncoding RNAs in Plants.

[3] Bhatia G., Goyal N., Sharma S., Upadhyay S.K., Singh K. (2017). Present Scenario of Long Non-Coding RNAs in Plants. Non-Coding RNA.

[4] Liu J., Wang H., Chua N.-H. (2015). Long noncoding RNA transcriptome of plants. Plant Biotechnol. J..

[5] Kim E.-D., Sung S. (2012). Long noncoding RNA: Unveiling hidden layer of gene regulatory networks. Trends Plant Sci..

[6] Zhang Y.-C., Liao J.-Y., Li Z.-Y., Yu Y., Zhang J.-P., Li Q.-F., Qu L.-H., Shu W.-S., Chen Y.-Q. (2014). Genome-wide screening and functional analysis identify a large number of long noncoding RNAs involved in the sexual reproduction of rice. Genome Biol..

[7] Chung P.J., Jung H., Jeong D.-H., Ha S.-H., Choi Y.D., Kim J.-K. (2016). Transcriptome profiling of drought responsive noncoding RNAs and their target genes in rice. BMC Genom..

[8] Singh U., Khemka N., Rajkumar M.S., Garg R., Jain M. (2017). PLncPRO for prediction of long non-coding RNAs (lncRNAs) in plants and its application for discovery of abiotic stress-responsive lncRNAs in rice and chickpea. Nucleic Acids Res..

[9] Lakra N., Kaur C., Anwar K., Singla-Pareek S.L., Pareek A. (2018). Proteomics of contrasting rice genotypes: Identification of potential targets for raising crops for saline environment. Plant Cell Environ..

[10] Kumari S., Sabharwal V.P.N., Kushwaha H.R., Sopory S.K., Singla-Pareek S.L., Pareek A. (2009). Transcriptome map for seedling stage specific salinity stress response indicates a specific set of genes as candidate for saline tolerance in *Oryza sativa* L.. Funct. Integr. Genom..

[11] Lakra N., Kaur C., Singla-Pareek S.L., Pareek A. (2019). Mapping the ‘early salinity response’ triggered proteome adaptation in contrasting rice genotypes using iTRAQ approach. Rice.

[12] Mishra M., Wungrampha S., Kumar G., Singla-Pareek S.L., Pareek A. (2021). How do rice seedlings of landrace Pokkali survive in saline fields after transplantation? Physiology, biochemistry, and photosynthesis. Photosynth. Res..

[13] Shin S.-Y., Jeong J.S., Lim J.Y., Kim T., Park J.H., Kim J.-K., Shin C. (2018). Transcriptomic analyses of rice (Oryza sativa) genes and non-coding RNAs under nitrogen starvation using multiple omics technologies. BMC Genom..

[14] Borah P., Das A., Milner M.J., Ali A., Bentley A.R., Pandey R. (2018). Long Non-Coding RNAs as Endogenous Target Mimics and Exploration of Their Role in Low Nutrient Stress Tolerance in Plants. Genes.

[15] Niazi F., Valadkhan S. (2012). Computational analysis of functional long noncoding RNAs reveals lack of peptide-coding capacity and parallels with 3′ UTRs. RNA.

[16] Yu B., Shan G. (2016). Functions of long noncoding RNAs in the nucleus. Nucleus.

[17] Noh J.H., Kim K.M., McClusky W.G., Abdelmohsen K., Gorospe M. (2018). Cytoplasmic functions of long noncoding RNAs. Wiley Interdiscip. Rev. RNA.

[18] Statello L., Guo C.-J., Chen L.-L., Huarte M. (2021). Gene regulation by long non-coding RNAs and its biological functions. Nat. Rev. Mol. Cell Biol..

[19] Schrumpfová P.P., Fojtová M., Fajkus J. (2019). Telomeres in Plants and Humans: Not So Different, Not So Similar. Cells.

[20] Vaquero-Sedas M.I., Gámez-Arjona F.M., Vega-Palas M.A. (2011). *Arabidopsis thaliana* telomeres exhibit euchromatic features. Nucleic Acids Res..

[21] Vrbsky J., Akimcheva S., Watson J.M., Turner T.L., Daxinger L., Vyskot B., Aufsatz W., Riha K. (2010). siRNA–mediated methylation of Arabidopsis telomeres. PLoS Genet..

[22] Xing J., Liu H., Jiang W., Wang L. (2021). LncRNA-encoded peptide: Functions and predicting methods. Front. Oncol..

[23] Kung J.T.Y., Colognori D., Lee J.T. (2013). Long Noncoding RNAs: Past, Present, and Future. Genetics.

[24] Zhang X., Wang W., Zhu W., Dong J., Cheng Y., Yin Z., Shen F. (2019). Mechanisms and Functions of Long Non-Coding RNAs at Multiple Regulatory Levels. Int. J. Mol. Sci..

[25] Misiak B., Ricceri L., Sąsiadek M.M. (2019). Transposable elements and their epigenetic regulation in mental disorders: Current evidence in the field. Front. Genet..

[26] Fan Y., Zhang F., Xie J. (2022). Overexpression of miR5505 enhanced drought and salt resistance in rice (*Oryza sativa*). bioRxiv.

[27] Parmar S., Gharat S.A., Tagirasa R., Chandra T., Behera L., Dash S.K., Shaw B.P. (2020). Identification and expression analysis of miRNAs and elucidation of their role in salt tolerance in rice varieties susceptible and tolerant to salinity. PLoS ONE.

[28] Arshad M., Gruber M.Y., Wall K., Hannoufa A. (2017). An Insight into microRNA156 Role in Salinity Stress Responses of Alfalfa. Front. Plant Sci..

[29] Goswami K., Mittal D., Gautam B., Sopory S.K., Sanan-Mishra N. (2020). Mapping the Salt Stress-Induced Changes in the Root miRNome in Pokkali Rice. Biomolecules.

[30] Lan T., Zheng Y., Su Z., Yu S., Song H., Zheng X., Lin G., Wu W. (2019). OsSPL10, a SBP-box gene, plays a dual role in salt tolerance and trichome formation in rice (*Oryza sativa* L.). G3 Genes Genomes Genet..

[31] Hou H., Jia H., Yan Q., Wang X. (2018). Overexpression of a SBP-Box Gene (VpSBP16) from Chinese Wild Vitis Species in Arabidopsis Improves Salinity and Drought Stress Tolerance. Int. J. Mol. Sci..

[32] Sharma A., Shahzad B., Rehman A., Bhardwaj R., Landi M., Zheng B. (2019). Response of Phenylpropanoid Pathway and the Role of Polyphenols in Plants under Abiotic Stress. Molecules.

[33] Tiwari S., Lata C., Chauhan P.S., Prasad V., Prasad M. (2017). A Functional Genomic Perspective on Drought Signalling and its Crosstalk with Phytohormone-mediated Signalling Pathways in Plants. Curr. Genom..

[34] Tiwari S., Nutan K.K., Deshmukh R., Sarsu F., Gupta K.J., Singh A.K., Singla-Pareek S.L., Pareek A. (2022). Seedling-stage salinity tolerance in rice: Decoding the role of transcription factors. Physiol. Plant.

[35] Zhang Z., Xu Y., Yang F., Xiao B., Li G. (2021). RiceLncPedia: A comprehensive database of rice long non-coding RNAs. Plant Biotechnol. J..

[36] Delgado C., Mora-Poblete F., Ahmar S., Chen J.-T., Figueroa C.R. (2021). Jasmonates and Plant Salt Stress: Molecular Players, Physiological Effects, and Improving Tolerance by Using Genome-Associated Tools. Int. J. Mol. Sci..

[37] Riyazuddin R., Verma R., Singh K., Nisha N., Keisham M., Bhati K.K., Kim S.T., Gupta R. (2020). Ethylene: A Master Regulator of Salinity Stress Tolerance in Plants. Biomolecules.

[38] Mittler R. (2017). ROS are good. Trends Plant Sci..

[39] Lim C., Kang K., Shim Y., Sakuraba Y., An G., Paek N.-C. (2020). Rice Ethylene Response Factor 101 Promotes Leaf Senescence through Jasmonic Acid-Mediated Regulation of *OsNAP* and *OsMYC2*. Front. Plant Sci..

[40] Tiwari S., Muthamilarasan M., Lata C. (2021). Genome-wide identification and expression analysis of Arabidopsis GRAM-domain containing gene family in response to abiotic stresses and PGPR treatment. J. Biotechnol..

[41] Tiwari S., Shweta S., Prasad M., Lata C. (2020). Genome-wide investigation of GRAM-domain containing genes in rice reveals their role in plant-rhizobacteria interactions and abiotic stress responses. Int. J. Biol. Macromol..

[42] Kong L., Zhang Y., Ye Z.-Q., Liu X.-Q., Zhao S.-Q., Wei L., Gao G. (2007). CPC: Assess the protein-coding potential of transcripts using sequence features and support vector machine. Nucleic Acids Res..

[43] Bastian M., Heymann S., Jacomy M. (2009). Gephi: An Open Source Software for Exploring and Manipulating Networks. Proc. Int. AAAI Conf. Web Soc. Media.

[44] Ge S.X., Jung D., Yao R. (2020). ShinyGO: A graphical gene-set enrichment tool for animals and plants. Bioinformatics.

[45] Jain M., Nijhawan A., Tyagi A.K., Khurana J.P. (2006). Validation of housekeeping genes as internal control for studying gene expression in rice by quantitative real-time PCR. Biochem. Biophys. Res. Commun..

[46] Livak K.J., Schmittgen T.D. (2001). Analysis of relative gene expression data using real-time quantitative PCR and the 2^−ΔΔCT^ Method. Methods.

[47] Saeed A.I., Sharov V., White J., Li J., Liang W., Bhagabati N., Braisted J., Klapa M., Currier T., Thiagarajan M. (2003). TM4: A Free, Open-Source System for Microarray Data Management and Analysis. Biotechniques.

