# Supplementary material for: Pokkali: A Naturally Evolved Salt-Tolerant Rice Shows a Distinguished Set of lncRNAs Possibly Contributing to the Tolerant Phenotype
Source: Int J Mol Sci. 2023 Jul 20;24(14):11677. doi: 10.3390/ijms241411677 (PMC10380863; doi:10.3390/ijms241411677)
Supplement: Supplementary file 1 [file ijms-24-11677-s001.zip › Figures S1-S7.pdf]

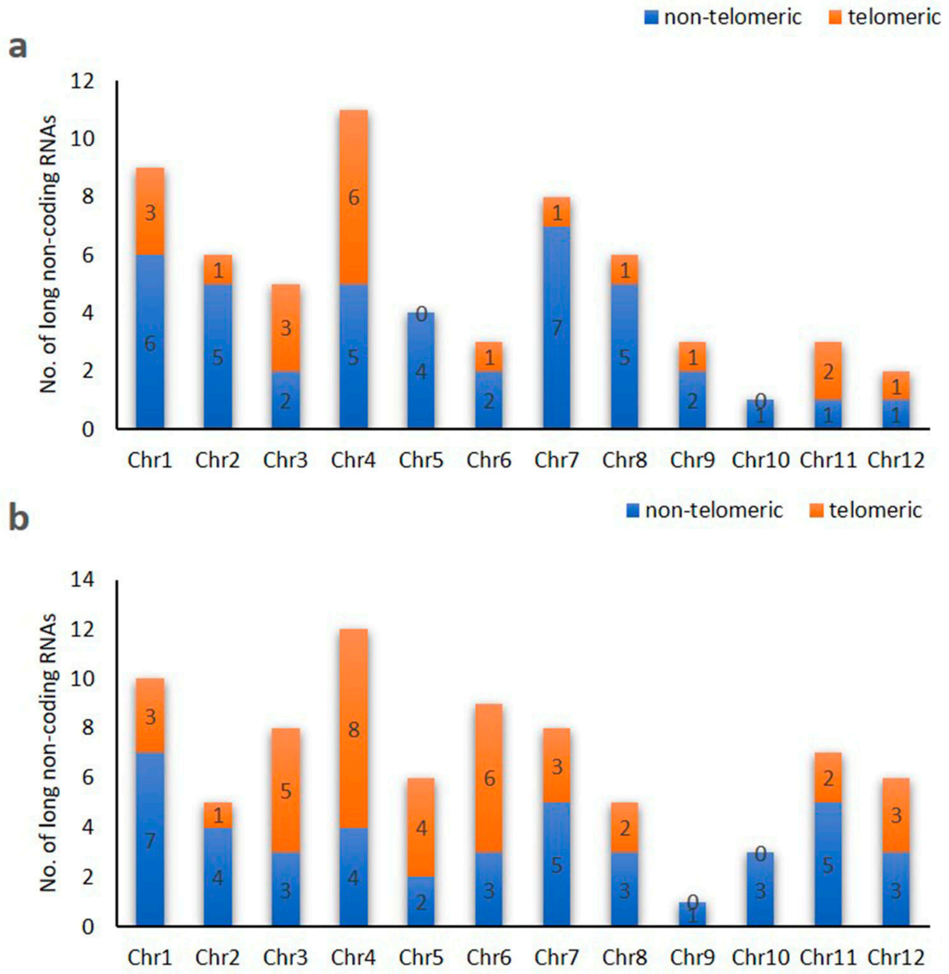

Figure S1: Figure depicts the number of lncRNAs present on the telomeric region of each chromosome in rice genotype (**a**) IR64 and (**b**) Pokkali.

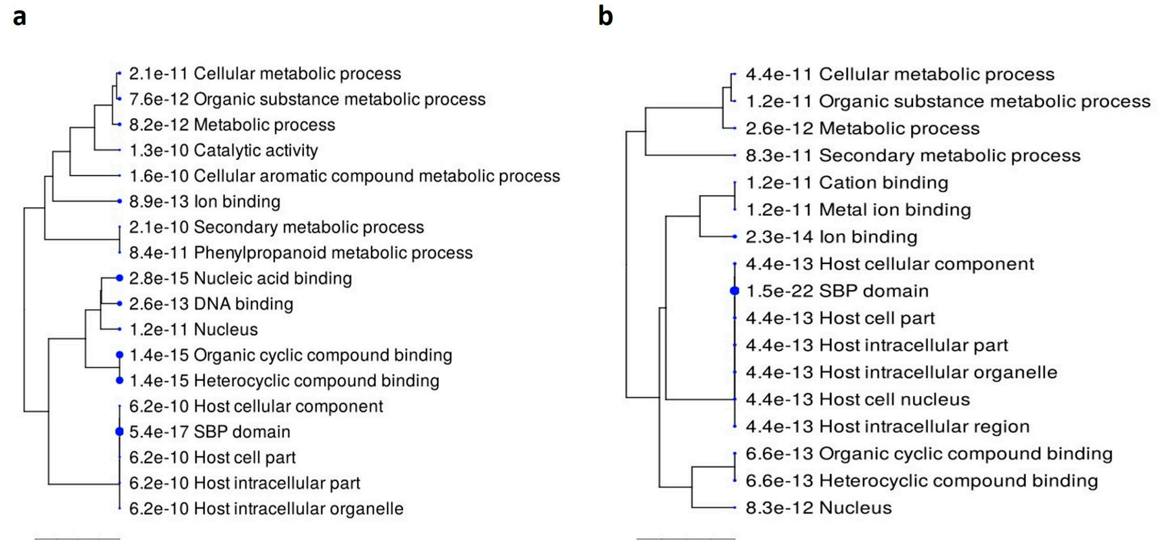

Figure S2: A hierarchical clustering tree summarizes the correlation among significant pathways in (**a**) IR64 and (**b**) Pokkali. Pathways with many shared genes are clustered together. Bigger dots indicate more significant *p*-values.

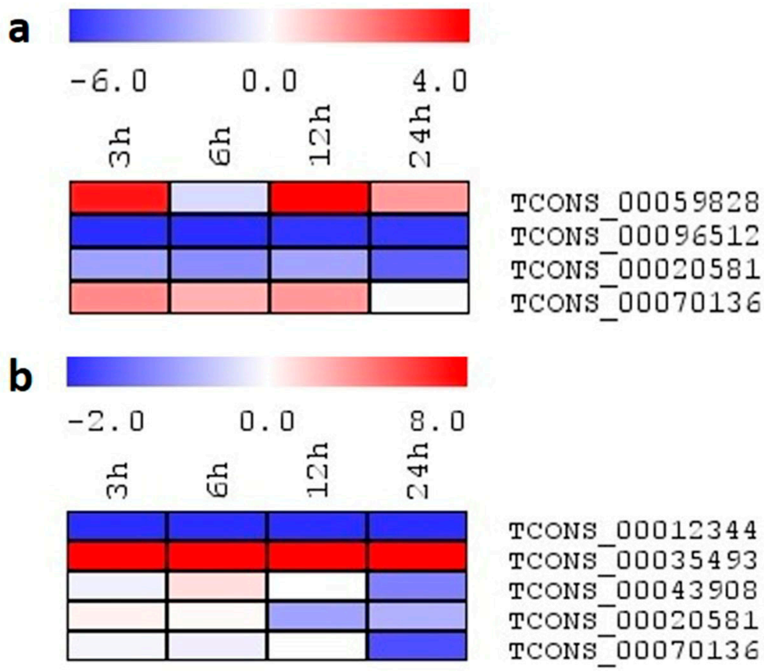

Figure S3 (a,b): The heatmap has been generated based on the log2 fold-change values showing differential expression of lncRNAs in rice genotypes (**a**) IR64 and (**b**) Pokkali exposed to ABA at 0, 3, 6, 12, and 24 h.

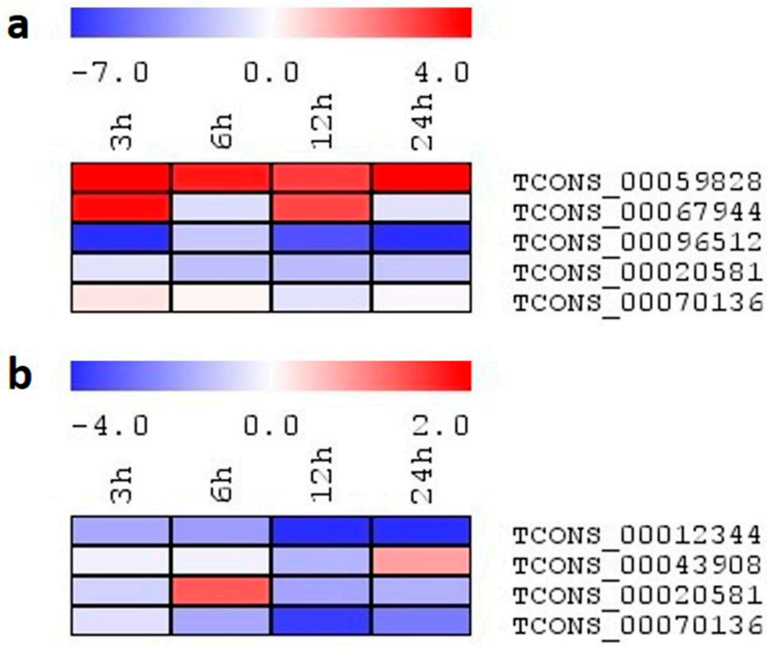

Figure S4 a,b): The heatmap has been generated based on the log2 fold-change values showing differential expression of lncRNAs in rice genotypes (**a**) IR64 and (**b**) Pokkali exposed to SA at 0, 3, 6, 12, and 24 h.

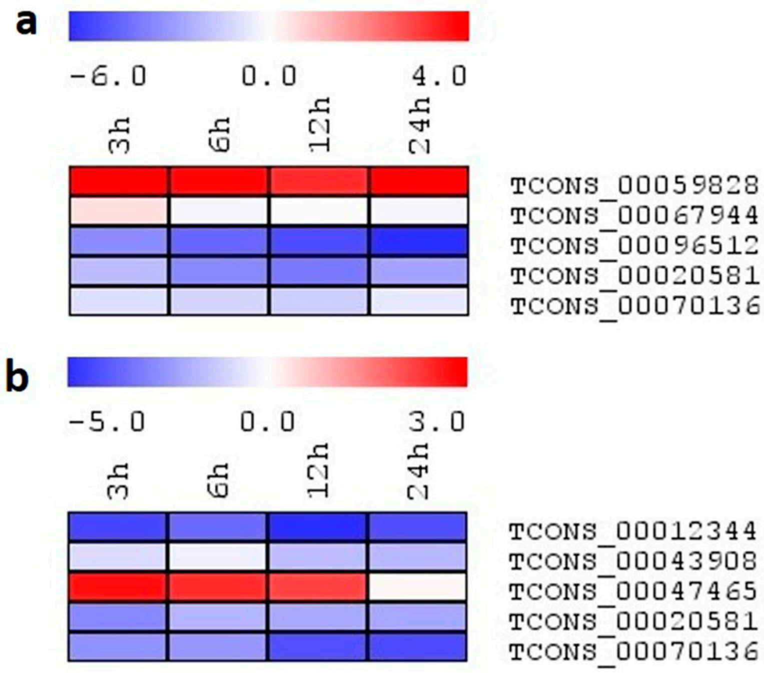

Figure S5 (a,b): The heatmap has been generated based on the log2 fold-change values showing differential expression of lncRNAs in rice genotypes (**a**) IR64 and (**b**) Pokkali exposed to JA at 0, 3, 6, 12, and 24 h.

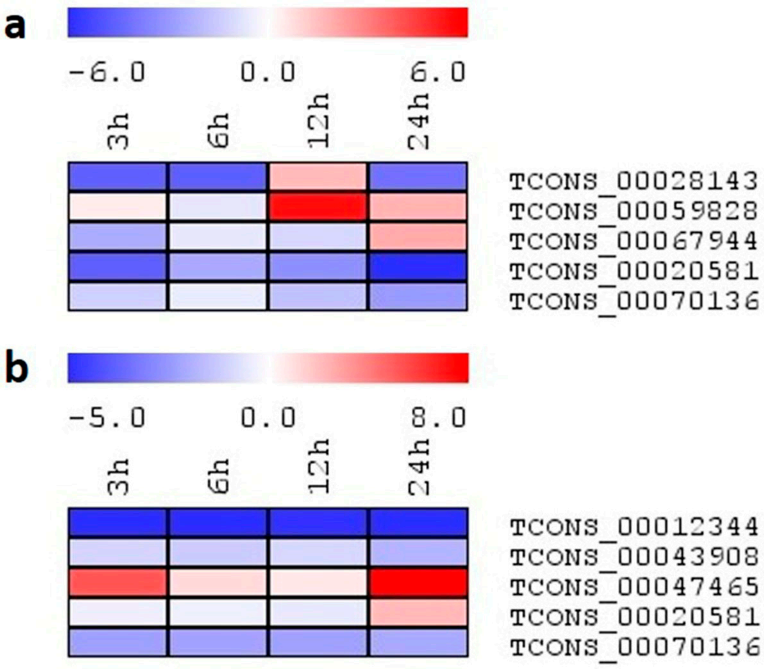

Figure S6 (a,b): The heatmap has been generated based on the log2 fold-change values showing differential expression of lncRNAs in rice genotypes (**a**) IR64 and (**b**) Pokkali exposed to ET at 0, 3, 6, 12, and 24 h.

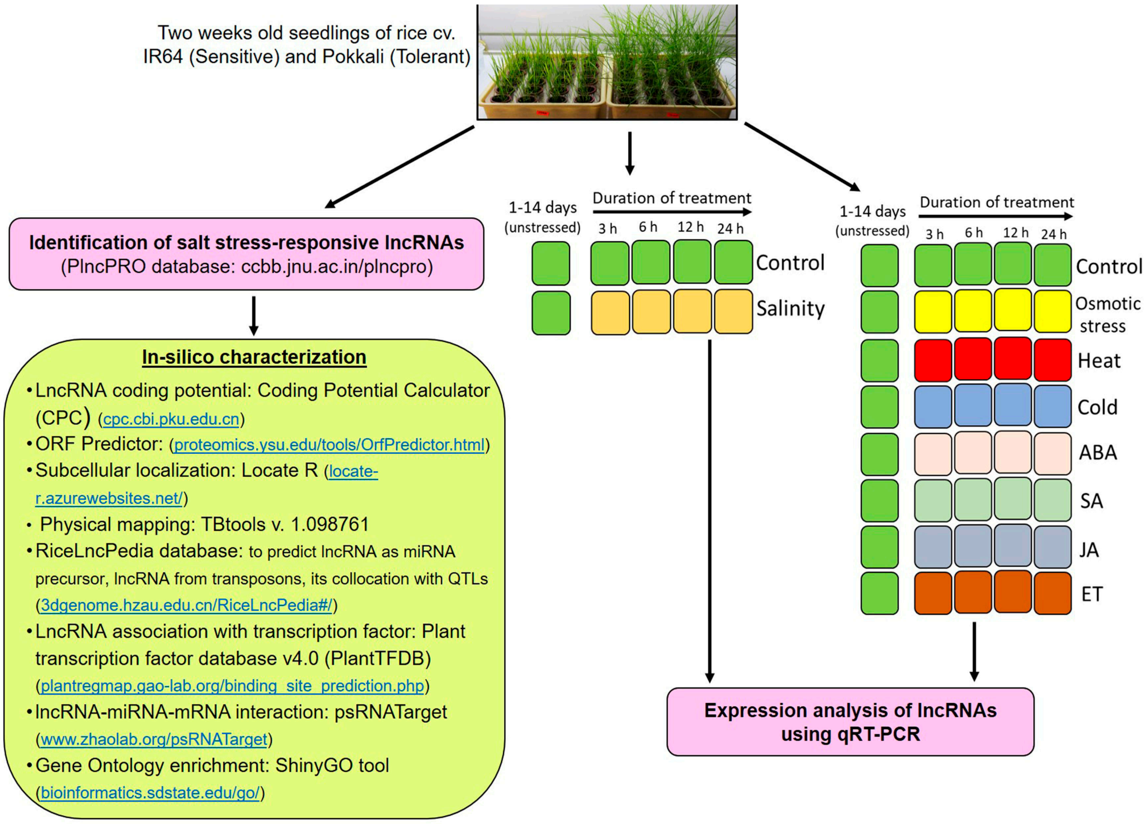

Figure S7: Schematic representation of the workflow and steps involved in the experiment.
