# Supplementary material for: Pokkali: A Naturally Evolved Salt-Tolerant Rice Shows a Distinguished Set of lncRNAs Possibly Contributing to the Tolerant Phenotype
Source: Int J Mol Sci. 2023 Jul 20;24(14):11677. doi: 10.3390/ijms241411677 (PMC10380863; doi:10.3390/ijms241411677)
Supplement: Supplementary file 1 [file ijms-24-11677-s001.zip › Table S1.pdf]

Table S1: List of the primers used in the study.

Cultivar	LncRNA ID	Oligos	References
Pokkali	T_00010836_F	CTGCGACATGTGCTTAGT	This study
	T_00010836_R	CCTGTTGTTCATGTCTAGGG	
	T_00012344_F	TACTCCCTCAACCTCCATAG	"
	T_00012344_R	GCAGGGTGGAATAGTGTTAC	
	T_00014021_F	GACACGACACATTGAGGTAG	"
	T_00014021_R	CCTCATCGGCATCTTCTTAC	
	T_00019521_F	CTCAACCTCCTCTCCATTTC	"
	T_00019521_R	GCTCAATCAGCTCCAAGAA	
	T_00020593_F	ATTCCTCAATCGCCTCCT	"
	T_00020593_R	GACGAAGCTGCCATTAATTTG	
	T_00035491_F	GAGGAACAGTACGTGCAATA	"
	T_00035491_R	TCTGCTACTTCTCGGAGTTA	
	T_00035493_F	CCACATCAGACAGGTTCAG	"
	T_00035493_R	GCAATGCAACTGCTCATC	
	T_00038079_F	GTGACCTGCTGATTGATGTT	"
	T_00038079_R	TCACCATGGCCTTCATTTG	
	T_00043908_F	ATCGTGTGCTCAATGGATAG	"
	T_00043908_R	GGGCGTATGAGAAAGAAGAG	
	T_00047465_F	TCACAACTCCTCGGTACAA	"
	T_00047465_R	TTCGCTTAGCCATGCTTAC	
	T_00070178_F	TTTGAGAGTGGTCTGGTTTG	"
	T_00070178_R	GGTAGTAGGCTCACATCAAAG	
	T_00079037_F	GGGTGTCCAGAGAAATGTTG	"
	T_00079037_R	TGTCAGTGTTCATGGATTGG	
	T_00087936_F	CCAACTTTGACTATCCGTCT	"
	T_00087936_R	CGTTTGACCGTCCGTCTT	
	T_00088033_F	TGGAAAGTACCCGACTAGAG	"
	T_00088033_R	GGCTGTCTATGCCTTGTTT	
	T_00104482_F	AGGCAGGTTGAAGGAGAT	"
	T_00104482_R	ACACGCTTGTGCTTTGT	
	T_00103084_F	TACATCGTCCGGCTCAA	"
	T_00103084_R	CATCTCGGTGCAAGCAA	
IR64	T_00002159_F	GACAAGTAACCTGCCAGATAG	"
	T_00002159_R	GTTCCCTGAATCTGCTTGT	
	T_00003058_F	TTCCTCTTCCTCTCCCTATC	"
	T_00003058_R	CTGTAGCTCACCGATGATTC	
	T_00013994_F	AACAGCCAAAGAATAAGCCA	"
	T_00013994_R	GTAGATTTGTTCGTAGCGACTT	
	T_00028143_F	GCTGAGTCGCTGATGATATG	"
	T_00028143_R	CCACACCAGCCAAGAAAT	

	T_00035411_F	GCAGGGAAAATGTGTGCT	"
	T_00035411_R	TGTTGAGCAAGAAGCTCACT	
	T_00035427_F	GCGTGGTAGGTACGAAATG	"
	T_00035427_R	CTCACTGCGCATATCTCTTT	
	T_00038200_F	ATAGCCATGTCTCCCTTCT	"
	T_00038200_R	CGGATGTGTATGGAGGAAAG	
	T_00059595_F	TTGGAGGAGGGAGAAAGAG	"
	T_00059595_R	CATCTACGCCTTCCATTCTG	
	T_00059828_F	TTCGGTTTGTAAGACTTCTGG	"
	T_00059828_R	CCCAACAATCAACCACAATAAC	
	T_00061576_F	CATCTACCCAGCAGGAGATA	"
	T_00061576_R	TGGACAATTTCAGGGACAAG	
	T_00067944_F	TCGTATCTTAGGTGGAGTTGA	"
	T_00067944_R	ATCTGCTGAGCTTGTTGTAG	
	T_00085253_F	CTCCCAACCCAATCTCTAATC	"
	T_00085253_R	CGACAACTTCCCTGTCATC	
	T_00096512_F	GGTGATTCCTACTCCTGAAG	"
	T_00096512_R	GTCAAATTATGCAAGGGTGAG	
	T_00096533_F	CAACTCACACGTCGAGATA	"
	T_00096533_R	GTCCACCAAAATTCATGTTC	
	T_00110813_F	CGTATTAGTTCATCGCGTAGG	"
	T_00110813_R	AGGGATGATTGCTTGATTGG	
Common in both cultivars	T_00020581_F	CGGTCGTACTCGTATGTTCT	"
	T_00020581_R	GGAACAGTACGGAAGAAATGG	
	T_00068689_F	CCCAGCAAGGTCAAATGT	"
	T_00068689_R	GGAGGTCGAGGGAATAGTAA	
	T_00046718_F	GCCGCGAGGATTAATTGAAG	"
	T_00046718_R	CACAAACCAAACCAACCCA	
	T_00070136_F	GTTGCTGATCCATTAGGTAGAC	"
	T_00070136_R	CAACGTGTCTGCATCCTATT	
Reference gene	Rice_UBQ5_F	ACCACTTCGACCGCCACTACT	[40]
	Rice_UBQ5_R	ACGCCTAAGCCTGCTGGTT	
